# A rare case of strangulated femoral hernia in a male child: Case report

**DOI:** 10.1016/j.ijscr.2024.110518

**Published:** 2024-10-24

**Authors:** Yilkal Teshome Numaro, Molla Asnake Kebede, Alemayehu Beharu Tekle, Michael Hawlet Tesfaye, Tariku Yigremachew Gossaya, Adefris Getachew Techane

**Affiliations:** a Department of Surgery, Gebre Tsadik Shawo General Hospital, Bonga, Ethiopia; b Department of Medicine, School of Medicine, College of Medicine and Health Sciences, Mizan - Tepi University, Mizan-Teferi, Ethiopia.; c Department of Emergency and Critical care medicine, School of Medicine, College of Medicine and Health Sciences, Mizan - Tepi University, Mizan-Teferi, Ethiopia; d Department of Pediatrics, School of Medicine, College of Medicine and Health Sciences, Wolkite University, Wolkite, Ethiopia; e Department of pediatrics, School of Medicine, College of Medicine and Health Sciences, Mizan - Tepi University, Mizan-Teferi, Ethiopia; f Department of Gynacology, School of Medicine, College of Medicine and Health Sciences, Mizan - Tepi University, Mizan-Teferi, Ethiopia

**Keywords:** Pediatric, Femoral hernia, Strangulated hernia, Groin swelling

## Abstract

**Background and importance:**

Strangulated femoral hernias are rare in pediatric patients. This case highlights the importance of early suspicion and diagnosis to prevent complications.

**Case presentation:**

A 14-years old male presented to our emergency department with a two-days history of worsening left groin pain. Clinical examination confirmed the diagnosis of a strangulated femoral hernia. A hernia sac was identified and the content was necrotic adipose tissue. Resection and hernia repair was performed successfully.

**Clinical dissection:**

Femoral hernia is an uncommon surgical entity in the pediatric population, and its diagnosis remains a challenge, with an incidence ranging from 0.3 % to 1 %. Unlike in adults, the incidence rates are similar by sex, though, like adults, right-side hernias are more common. However, our case presents with a left-side hernia.

**Conclusion:**

Strangulated femoral hernias should be considered in pediatric patients with acute groin pain and a history of reducible hernias. Early diagnosis and prompt surgical intervention are crucial to preventing complications.

## Highlights

• Femoral hernia is less common in children than in adults, but it can still occur.
• Femoral hernias in children can cause pain or discomfort in the groin area
• If left untreated, femoral hernias can lead to serious complications
• Treatment typically involves surgical repair to strengthen the weakened area of the abdominal wall

## 1 Introduction

Femoral hernias rarely occur in children, with an incidence <1 % of all pediatric groin hernias [1,2]. Pre-operative misdiagnosis is common [3].

This has been attributed to their relative rarity and a clinical presentation that may mimic other groin pathologies such as inguinal hernia, lymphadenitis and encysted hydrocele [4]. As a result there is an increased incidence of complications such as strangulation and perforation of the small bowel [5].

In this case report we reported a case of strangulated femoral hernia in a 14 years old male child to highlight the importance of proper diagnosis and management as it is a rare occurrence.

## 2 Case presentation

The case was a 14 years old male child who presented with colicky abdominal pain of two days duration. Associated with this complaint he had two episodes of vomiting of ingested matter. He was able to pass feces and flatus and had no abdominal distension. He had history of left groin swelling that he noticed two years back which was small initially and spontaneously reducible. For the past two days the swelling increased in size, became tender and irreducible.

**Fig. 1:**
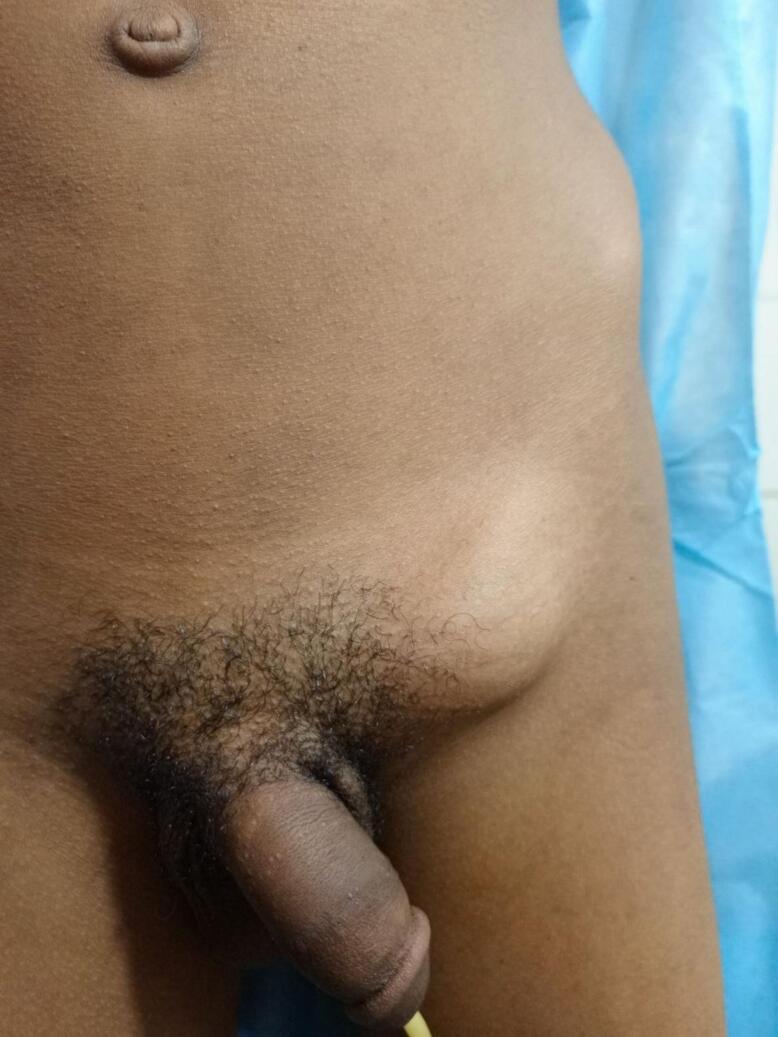
Left Femoral hernia.

He was tachycardic (120), had normal Blood pressure and no fever. His conjunctiva was pink. On Abdominal examination, it was not distended, soft abdomen that moves with respiration and normo active bowel sounds. He had left side tense tender 2.5 cm by 2.5 cm irreducible groin swelling below and lateral to pubic tubercle, it was non translucent and cough impulse was negative [Fig. 1]. Digital rectal examination (DRE) was normal. On Genitourinary system, he had normal scrotal growth and both testes were in anatomic positions.

**Fig. 2:**
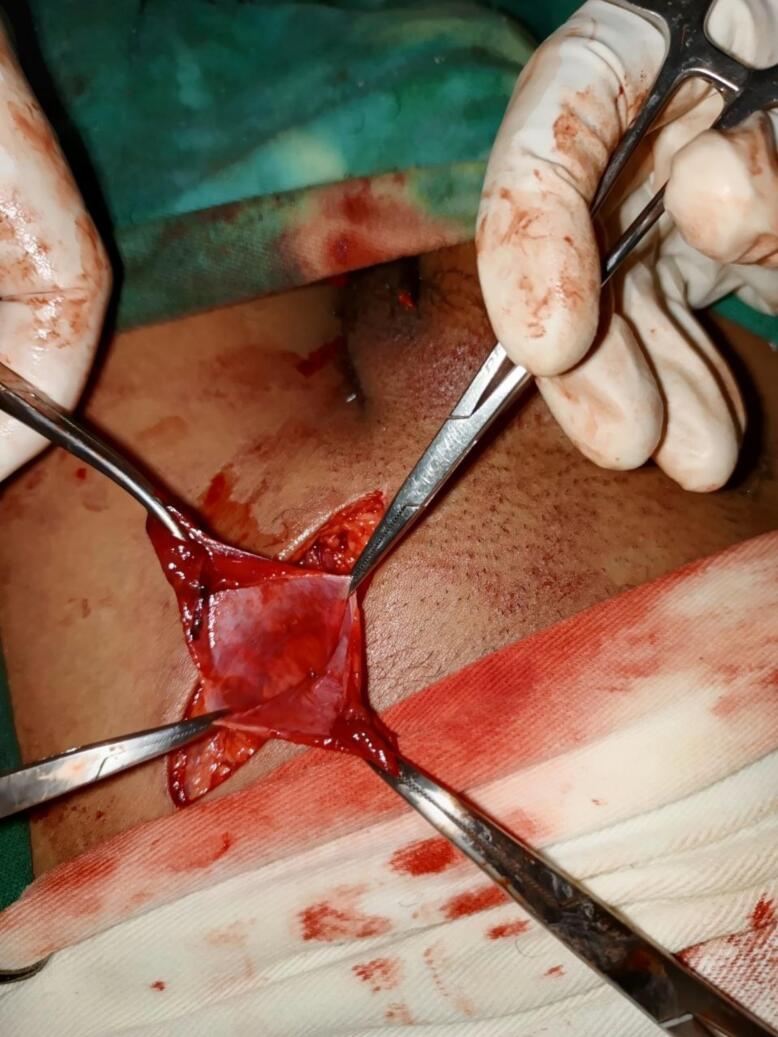
Empty hernia sac.

His complete blood count (CBC) was in normal range and Ultrasound which was done by a Medical Radiology technologist since there was no Radiologist available in our hospital revealed left side inguinal defect with bowel content and fluid protruding through a neck of 1.3 cm with an index of left incarcerated indirect inguinal hernia, but clinical examination showed the swelling was located below and lateral to the pubic tubercle. With the preoperative (Preop) diagnosis of strangulated femoral hernia, Emergency exploration through a femoral approach proved to be typical femoral hernia with a narrowed neck in the femoral ring. A hernia sac was identified and inspected [Fig. 2] and the content was necrotic adipose tissue.

**Fig. 3:**
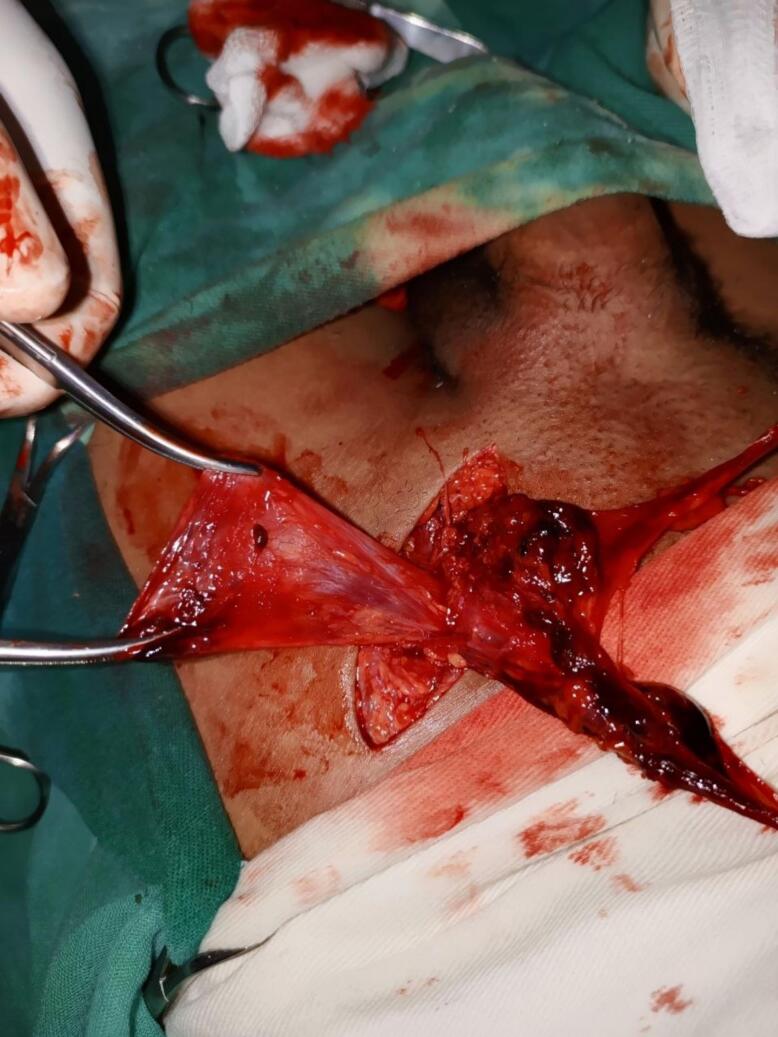
Necrotic adipose tissue.

**Fig. 4:**
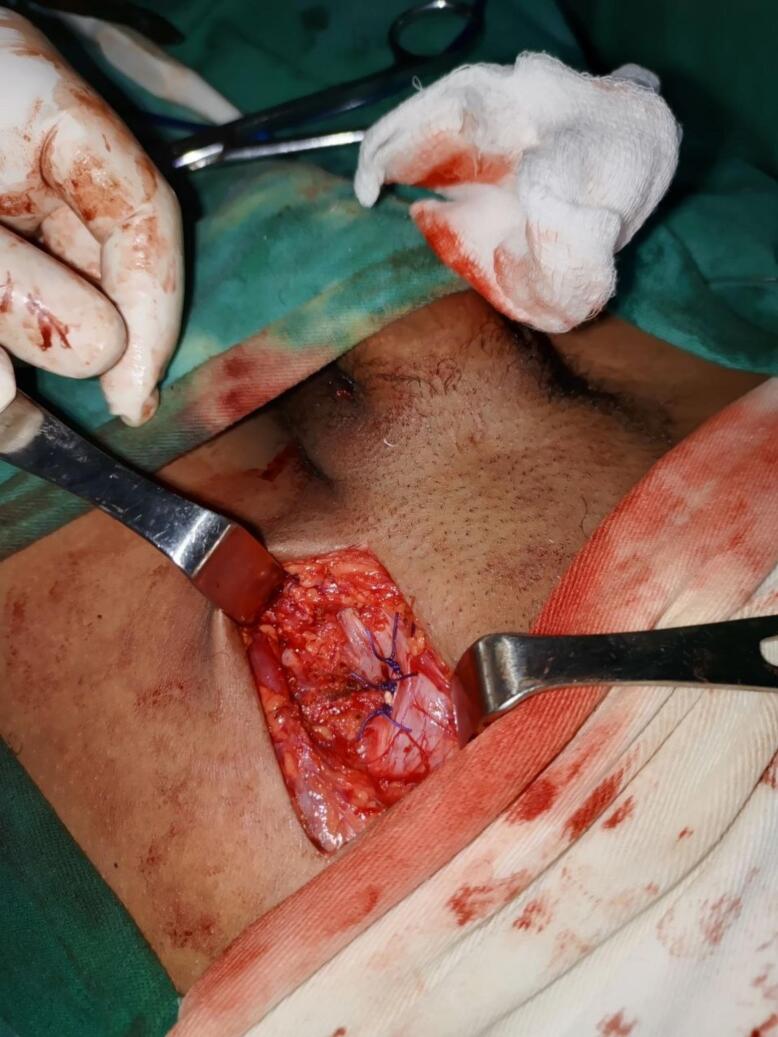
Mcvay closure of the Femoral ring.

The dark necrotic adipose tissue was excised [Fig. 3] the hernia sac was closed using a high purse string ligature and then resected. The Femoral ring was closed using McVay procedure and absorbable vicryl suture material was used [Fig. 4]. The wound closed in layers. The post operative course was uneventful and child discharged from hospital three days later. He was examined at the referral clinic on his 14th postoperative day, he started on regular diet, no subjective complaint, the wound site healed and there were no complications. Further out patient follow up done at 6 weeks and 3 months following the surgery, the patient remained stable and he resumed normal activities.

## 3 Discussions

Femoral hernia is an uncommon surgical entity in the pediatric population and the diagnosis remains a challenge [6]. with an incidence ranges 0.3–1 % [7,8,9]. Distinct from adults, incidence rates were similar by sex but, like adults, right-sided hernias dominate [4]. unlike our case which presented with a left sided hernia. Femoral hernia are rarely seen in infancy, accounting for <10 % of all cases [8,9].

Although the etiology of femoral hernia is still imprecise, McVay and Savage had proposed the hypothesis that a congenitally narrow insertion of the posterior inguinal wall on to Cooper's ligament results in enlargement of the femoral ring and predisposes to herniation in the event of high intra-abdominal pressure [10]. However, a large series of post-mortem examination did not reveal congenital femoral hernia sac [11]. It has been reported on children who developed symptoms in the neonatal period that led to the thought that there must be some congenital anatomic causal factors [8].

Femoral hernias are only appropriately diagnosed in 53 % of cases. Thus, they are sometimes managed as regular inguinal hernias with open approaches [2,12].

In retrospective study done at Royal Hospital for Sick Children, Sciennes Road, Edinburgh EH9 1LF, UK and Ninewells Hospital and Medical School, Dundee DD1 9SY, UK with pediatrics femoral hernia over a 12-year period, sixteen children with a median age of 7 (range 3–16) years were identified and they concluded Femoral hernia are a diagnostic challenge and a high index of clinical suspicion is necessary. The long-term results of pediatrics femoral hernia surgery are excellent [3].

In a retrospective study done at Children's Research Centre, Our Lady's Hosp. for Sick Children, University College Dublin, Crumlin, 12 Dublin, Ireland of all patients who underwent femoral hernia repair between 1980 and 2000. Thirty-eight children (20 females, 18 males) with the median age of 5.5 years. This study shows that femoral hernia is still a commonly misdiagnosed condition. A correct preoperative diagnosis will lead to appropriate surgical management, thus avoiding unnecessary morbidity and preventing unnecessary reoperations [2].

In a study done at Div. of Pediatric General Surgery, IWK Grace Health Centre, Dalhousie University, Halifax, NS, Canada and Div. of Pediatric General Surgery, IWK Grace Health Centre, Dalhousie University, 5850 University Ave, Halifax, NS B3J 3G9, Canada. They reviewed all patients under 15 years of age with femoral hernia (January 1977 to January 1998). There were nine girls (53 %) and eight boys (47 %). Age range was 2 to 15 years. They concluded Femoral hernia in childhood is a challenging clinical problem because of its rarity and similar clinical presentation as indirect inguinal hernia [7].

Incorrect diagnosis and treatment may be complicated by necrosis and perforation of the intestines with increased morbidity [4]. Several authors reported strangulation rates of 10 % to 40 % for femoral hernias in the pediatric group [13]. this is similar to our case which presented with strangulation and necrotic adipose tissue. Femoral hernia diagnosis is mainly made by careful physical examination of soft and non-tender protrusion in the femoral triangle [14]. It presents as a bulge below and lateral to the pubic tubercle, in a location inferior and lateral to that of the frequently occurring indirect hernia, and which is not controlled by digital occlusion of the internal inguinal ring [4,[15], [16], [17]] as this was the typical presentation in our case.

As in our case, Appropriate femoral hernia diagnosis is made by careful medical history taking and clinical examination [7]. A considerable proportion of patients with signs evocative of hernia are found to have confusing physical examination findings. In this patient group, ultrasound can be employed as a safe and non-invasive diagnostic tool to confirm the femoral hernia diagnosis and show the hernia content [9,14,18]. In this case ultrasound was not confirmatory. The methods of femoral hernia management in children are varied, ranging from simple dissection and ligation of the hernial sac to laparoscopic repair and mesh-plug occlusion of the femoral canal [12,19].

In most series, pediatric femoral hernias are managed using the conventional McVay procedure, in which femoral ring closure is carried out by the apposition of the transversal fascia and Cooper's ligament through the open anterior approach [6] and our patient underwent femoral hernia repair using McVay procedure. The work has been reported in line with the SCARE criteria [20].

## 4 Conclusion

Femoral hernia is a rare and diagnostically challenging condition in childhood. Delayed or incorrect diagnosis can lead to significant morbidity. Most cases can be accurately diagnosed through careful clinical examination. This case study suggests that if every groin lump in a child is regarded with a high index of suspicion, it can avoid the morbidity and complication associated with the misdiagnosis.

## Informed consent

Written informed consent was obtained from the patient's parents for publication and any accompanying images. A copy of the written consent is available for review by the Editor-in-Chief of this journal on request.

## Ethical approval

Ethical approval for this study was obtained from the hospital management. Reference no: BHS/0132/2016.

## Guarantor

Molla Asnake Kebede.

## Research registration number

Not applicable.

## Funding

The case report, authorship, and/or publication of this work were done without outside funding.

## Author contribution

Yilkal Teshome Numaro, MD.

Play major role in patient management. Involved in the conception and design of the study, drafting and revising of the article and final approval of the version to be submitted and also involved in direct management of the patient.

Molla Asnake Kebede, MD.

Involved in the conception and design of the study, drafting and revising of the article and final approval of the version to be submitted and also involved in direct management of the patient.

Alemayehu Beharu Tekle, MD.

Involved in the conception and design of the study, drafting and revising of the article and final approval of the version to be submitted and also involved in direct management of the patient.

Tariku Yigremachew Tesfaya.

Involved in the conception and design of the study, drafting and revising of the article and final approval of the version to be submitted and also involved in direct management of the patient.

Michael Hawlet Tesfaye.

Involved in the conception and design of the study, drafting and revising of the article and final approval of the version to be submitted and also involved in direct management of the patient.

Adefris Getachew Techane

Involved in the conception and design of the study, drafting and revising of the article and final approval of the version to be submitted and also involved in direct management of the patient.

## Conflict of interest statement

The authors report no conflicts of interest in this work.

## Data Availability

On a valid request, the corresponding author will provide access to the datasets that were gathered and used to conduct this article.
